# Editorial Comment: Long-term effectiveness and complication rates of bladder augmentation in patients with neurogenic bladder dysfunction: A systematic review

**DOI:** 10.1590/S1677-5538.IBJU.2021.01.04

**Published:** 2020-11-18

**Authors:** Marcio Augusto Averbeck

**Affiliations:** 1 Hospital Moinhos de Vento Unidade de Videourodinâmica Porto AlegreRS Brasil Chefe de Neuro-Urologia, Unidade de Videourodinâmica, Hospital Moinhos de Vento. Porto Alegre, RS, Brasil

Hoen L’ ^1^, Ecclestone H ^2^, Blok BFM ^1^, Karsenty G ^3^, Phé V ^4^ ^5^,Bossier R ^3^ et al.

## COMMENT

International guidelines support a timely diagnosis and an individualized treatment to prevent upper and lower urinary tract deterioration in neuro-urological patients (1, 2). Reconstructive surgery may be needed in high-risk patients, who failed conservative treatment for neurogenic detrusor overactivity (3). Hoen et al. performed a PRISMA systematic review to evaluate effectiveness and safety of bladder augmentation for adult neuro-urological patients. A total of 20 studies including 511 patients were eligible for inclusion. Primary outcomes were assessed in 16 of the 20 studies and showed improved quality of life and anatomical changes as well as stable renal function. Continence rates were reported in only 14 series. A total of 225 patients either were incontinent or had an indwelling catheter preoperatively. Only 30 patients were not completely continent postoperatively (87% success rate). Long-term complications continued up to 10 years postoperatively, including bowel dysfunction in 15% of the patients, stone formation in 10%, five bladder perforations and one bladder cancer. These outcomes reinforce both the role of bladder augmentation in high-risk neuro-urological patients and the importance of longer-term follow-up. However, the studies included in this systematic review had a low-level of evidence (mostly level 4; only one level 3 study). Therefore, further research initiatives should include structured quality of life assessments, detailed description of inclusion and exclusion criteria, surgical technique and post-operative complications. Perhaps an international registry/working group may overcome these inherent limitations and increase the level of evidence in the near future.
